# Reduced infectivity of waterborne viable but nonculturable *Helicobacter pylori* strain SS1 in mice

**DOI:** 10.1111/hel.12391

**Published:** 2017-04-24

**Authors:** Kevin F. Boehnke, Kathryn A. Eaton, Clinton Fontaine, Rebecca Brewster, Jianfeng Wu, Joseph N.S. Eisenberg, Manuel Valdivieso, Laurence H. Baker, Chuanwu Xi

**Affiliations:** ^1^ Department of Environmental Health Sciences University of Michigan Ann Arbor MI USA; ^2^ Department of Microbiology and Immunology University of Michigan Ann Arbor MI USA; ^3^ Department of Epidemiology University of Michigan Ann Arbor MI USA; ^4^ Division of Hematology and Oncology, Department of Internal Medicine University of Michigan Ann Arbor MI USA

**Keywords:** *Helicobacter pylori*, infectivity, SS1, waterborne transmission

## Abstract

**Background:**

*Helicobacter pylori* infection has been consistently associated with lack of access to clean water and proper sanitation, but no studies have demonstrated that the transmission of viable but nonculturable (VBNC) *H. pylori* can occur from drinking contaminated water. In this study, we used a laboratory mouse model to test whether waterborne VBNC *H. pylori* could cause gastric infection.

**Materials and Methods:**

We performed five mouse experiments to assess the infectivity of VBNC *H. pylori* in various exposure scenarios. VBNC viability was examined using Live/Dead staining and Biolog phenotype metabolism arrays. High doses of VBNC *H. pylori* in water were chosen to test the “worst‐case” scenario for different periods of time. One experiment also investigated the infectious capabilities of VBNC SS1 using gavage. Further, immunocompromised mice were exposed to examine infectivity among potentially vulnerable groups. After exposure, mice were euthanized and their stomachs were examined for *H. pylori* infection using culture and PCR methodology.

**Results:**

VBNC cells were membrane intact and retained metabolic activity. Mice exposed to VBNC *H. pylori* via drinking water and gavage were not infected, despite the various exposure scenarios (immunocompromised, high doses) that might have permitted infection with VBNC *H. pylori*. The positive controls exposed to viable, culturable *H. pylori* did become infected.

**Conclusions:**

While other studies that have used viable, culturable SS1 via gavage or drinking water exposures to successfully infect mice, in our study, waterborne VBNC SS1 failed to colonize mice under all test conditions. Future studies could examine different *H. pylori* strains in similar exposure scenarios to compare the relative infectivity of the VBNC vs the viable, culturable state, which would help inform future risk assessments of *H. pylori* in water.

## 1 Introduction

*Helicobacter pylori* (*H. pylori*) is a gastrointestinal bacterium that causes gastritis, peptic ulcers and, over time, gastric adenocarcinoma.^1^, ^2^ *Helicobacter pylori* infection is hypothesized to be transmitted through multiple routes, including vertically from mother to child and through contaminated reservoirs like food and water.^3^, ^4^ A body of evidence suggests that contaminated water may be a source of *H. pylori* infection, with epidemiological studies consistently associating *H. pylori* infection with lack of access to potable drinking water and proper sanitation.^3^, ^5^, ^6^, ^7^, ^8^, ^9^ Furthermore, *H. pylori* has been detected in water using various molecular biology techniques, such as quantitative polymerase chain reaction (qPCR) and microscopy methods,^5^, ^10^, ^11^, ^12^, ^13^ and there are reports that it has been cultured from water.^14^, ^15^, ^16^, ^17^ *Helicobacter pylori* enters a viable but not culturable (VBNC) state within a few days after inoculation into water.^18^, ^19^, ^20^ This change is often accompanied by a morphological change from a spiral bacillus to a U‐shaped or coccoid form, and *H. pylori* has been found in the VBNC state in all these morphologies in the natural environment.^18^, ^21^ However, although *H. pylori* has been cultured from wastewater and drinking water, it is unclear whether this was due to the culturable form being present in the water or investigators being able to revert the VBNC form back to a culturable form using appropriate media.

The fact that *H. pylori* is present in both a culturable and VBNC state has not been accounted for when assessing risk associated with waterborne *H. pylori*. For example, a risk model of waterborne *H. pylori* infection using a quantitative microbial risk assessment methodology^22^ did not consider the VBNC form of *H. pylori*. Likewise, our recent study showing that constant exposure to the viable, cultural form of *H. pylori* in drinking water can infect mice did not account for exposure to the VBNC form.^20^ While previous studies found that VBNC *H. pylori* administered via gavage could cause infection in mice,^19^, ^23^ the gavage exposure method is not representative of exposure to drinking water. To fill this gap in the literature, we examined the infectivity of the VBNC form of *H. pylori* in water.

## 2 Materials and Methods

### 2.1 Transmission and exposure groups

Our studies were carried out sequentially following our initial dosing experiments that examined the infectious dose of viable, culturable *H. pylori* in water.^20^ We performed four mouse experiments to assess the infectivity of VBNC *H. pylori* in various different exposure scenarios (Table 1). Concentrations of VBNC *H. pylori* were chosen based on previous studies^19^, ^20^, ^23^ and on the amounts of *H. pylori* found in sources of recreational and drinking water worldwide.^24^, ^25^ We first employed a classic single‐hit exposure model with waterborne VBNC *H. pylori*, examining whether a single day of water with a high dose of *H. pylori* could cause infection, choosing the high end of waterborne concentrations to test a “worst‐case” scenario and to try to ensure a higher chance of experimental infection; 4 weeks was chosen as the time to wait until euthanasia, given that She et al.^19^ had found slightly increased colonization rates at 4 weeks compared to 3 weeks. The sample size of 40 mice was chosen for consistency with our previous dosing experiments, in which each exposure group had 40 mice. When this failed to induce infection, we did two follow‐up experiments (Table 1, experiments 2 and 3). We increased the number of days of exposure (six instead of one), and also exposed severe combined immunodeficient mice to a single day of waterborne *H. pylori*, hypothesizing that more doses and immunocompromised hosts would be more likely to increase infection based on the results of our previous experiments.^20^ When these also failed to induce infection, we increased the exposure length again and increased the number of mice to 100 to increase the likelihood of seeing infection. In these experiments, we used a similar experimental design to our original dosing studies,^20^ exposing the mice to 56 days of contaminated water (experiment 4), and further decreasing the time until euthanasia. When this also failed to induce infection, we did a final follow‐up study in which we gavaged mice with four doses of ~2*10^8^ cells of VBNC SS1 over 2 weeks. This, too, failed to induce infection.

**Table 1:** Experimental overview of various drinking water exposure scenarios

Experiment number	Exposure groups	Time to VBNC conversion	Exposure	Euthanized
1	40 C57/BL6 mice (20 male, 20 female)	2 d	Exposure to 1 d of 10^9^ cells/L of VBNC *Helicobacter pylori*	4 wk after final exposure
2	40 C57/BL6 mice (20 male, 20 female)	2 d	Exposure to 6 d of 10^9^ cells/L of VBNC *H. pylori* a	2 wk after final exposure
3	10 C57/BL6 Severe Combined Immunodeficient mice (4 male, 6 female)	2 d	Exposure to 1 d of 10^9^ cells/L of VBNC *H. pylori*	1 wk after final exposure
4	100 C57/BL6 mice (50 male, 50 female)	4 d	Consistent exposure to >10^9^ cells/L of VBNC *H. pylori* over 56 d	4 d after final exposure
Negative control	10 C57/BL6 mice (4 male, 6 female)	N/A	Sterile, filtered tap water for 60 d	Day 60
Positive control	10 C57/BL6 mice (4 male, 6 female)	N/A	Consistent exposure to >10^9^ cells/L of viable, culturable *H. pylori* over 56 d	4 d after final exposure

a Mice were exposed to contaminated drinking water for 3 d, followed by 11 d of sterile water, and then another 3 d of contaminated water.

The mice were exposed to water contaminated with ~10^9^ cells/L VBNC *H. pylori* (See Table 1). In experiments 1‐3, contaminated water was removed after 24 hours and replaced with either a bottle of freshly contaminated water or (when appropriate) sterilized, filtered tap water. Each exposure group had 20 cages, with two mice per cage per the Animal Care and Use Committee regulations. In experiment 4, water was changed twice per week, every 3‐4 days. As a negative control, 10 mice (five cages) were given sterile, filtered tap water for 9 weeks. As a positive control, 10 mice (five cages) were given sterile, filtered tap water inoculated with viable, culturable *H. pylori* for 9 weeks. All mice were housed at University Laboratory Animal Medicine facilities at the University of Michigan Medical School, and all experiments were approved by the Animal Care and Use Committee.

### 2.2 Bacterial strain

SS1 (Sydney Strain 1) was selected for this study for consistency with our previous studies,^20^ and because it colonizes mice more successfully than other *H. pylori* strains.^26^

### 2.3 *H. pylori* cultivation, counting, and inoculation

*Helicobacter pylori* cultivation was carried out as previously described.^20^ Briefly, SS1 was plated from stocks and grown at 37°C on 5% sheep blood tryptic soy agar II plates (BBL, Franklin Lakes, NJ, USA) in microaerobic conditions. After 3 days, colonies were collected and suspended in plates of Brucella broth (Remel, Columbus, OH, USA) supplemented with 10% heat‐inactivated fetal bovine serum (Fisher Scientific, Waltham, MA, USA). After shaking overnight in microaerobic conditions at 37°C, the broth was centrifuged at 1917 *g* and 4°C for 20 minutes. The supernatant was removed, and the pellet was suspended in 1× PBS. To confirm the concentration of *H. pylori*, the stock suspension was serially diluted onto 5% sheep blood tryptic soy agar II plates (BBL). Sterilized, filtered tap water was then inoculated with the stock suspension. After 4‐7 days of growth, the number of *H. pylori* colonies was counted and the stock solution concentration was back‐calculated.

### 2.4 *H. pylori* viability in water

Previous methodologies for inducing the VBNC state have differed across studies. She et al.^19^ inoculated sterilized tap water with live *H. pylori* and stored it at 4°C for 40 days, defining cells as VBNC when they were in the coccoid state and did not grow when plated. Wang et al.^23^ incubated fresh *H. pylori* colonies in Ham's F12 medium with 10% calf serum for 3 days, then stored them at 4°C, defining cells as VBNC once they stopped growing. Cellini et al.^27^ inoculated a Brucella broth/2% fetal calf serum solution with fresh *H. pylori* and incubated it for 20 days until the cells no longer grew when plated. As we wanted to examine the infectivity of VBNC *H. pylori* in water, we chose to incubate *H. pylori* in water for the VBNC conversion.

Inoculated water was held for 2‐4 days at room temperature to ensure that the VBNC conversion had occurred before giving water to the mice. To check culturability, inoculated water was plated on 5% sheep blood tryptic soy agar II media and incubated for 7 days in microaerobic conditions at 37°C. *Helicobacter pylori* cells were checked for viability and morphology using microscopy at 60× magnification and Live/Dead BacLight Bacterial Viability Kit (Life Technologies, Eugene, OR, USA). To undertake viability and morphological analyses, 50 mL of water was centrifuged at 10 400 *g* for 3 minutes, the supernatant was removed, and cell pellets were suspended in BacLight Live/Dead dye. After incubating for a minimum of 15 minutes in the dark, the cell suspensions were examined in triplicate per the manufacturer's instructions.

Metabolic activity of VBNC *H. pylori* cells was examined using Biolog Phenotypic Microarray plates PM1, which contain 95 separate carbon sources which are commonly utilized by a variety of microbial species. All necessary reagents were purchased from Biolog (Hayward, CA, USA). *Helicobacter pylori* cells were grown on 5% Sheep blood Tryptic Soy Agar II media, then collected from the plates and suspended in sterile, autoclaved water. Cell suspensions were stored at room temperature for 0, 3, 4, 7, or 8 days. At each respective time point, cell suspensions were spun down at 1917 *g* for 20 minutes. The supernatant was discarded, and the resulting pellets were checked for metabolic activity using the PM1 plate. Briefly, pellets were re‐suspended in inoculating fluid IF‐0a GN/GP (1.2×), and then supplemented to a final concentration of 0.05% bovine serum albumin (BSA) and 1.25 mmol/L NaHCO_3_. (*H. pylori* has been shown to use more carbon sources and grow successfully in media containing BSA,^28^, ^29^ so it was included to ensure better visualization of metabolic activity in the VBNC state.) Dye mix D (Biolog) was then added to achieve a final concentration of 0.01%; 100 μL of this solution was pipetted into each well of the PM1 plate, which was then incubated in microaerobic conditions for 48 hours. Cells were considered to be metabolically active if they induced a color change in any of the wells containing nutrient sources, and the negative control had no color change. This was not measured in a quantitative way, but checked visually, as the purpose of this experiment was to examine *H. pylori* VBNC cell viability rather than examine the carbon sources used.

### 2.5 Dose estimation

To estimate the doses consumed by the mice, water bottles were weighed before being placed in cages and immediately after their removal. As water drips out of water bottles when they are placed in the cage and when the cages are moved, “dummy” bottles were filled with water and treated in the exact same way as experimental bottles. The amount of water lost from dummy bottles was averaged and that average was subtracted from the total water lost from each bottle. As mice were housed two per cage, the adjusted total per cage was then halved to provide the individual dose per mouse.

### 2.6 Mouse euthanasia, verification and quantification of infection

After exposure, the mice were euthanized and their stomachs were collected. The stomach was weighed, homogenized in 1× PBS, and serial dilutions of the homogenate were plated on *H. pylori* selective media (Columbia blood agar base with 10% horse blood, Dent Supplement, 300 mg/L urea, and 3500 U polymyxin B/L).^30^ Presumptive *H. pylori* isolates were counted and then checked for urease activity using urease indicator broth (0.33 mol/L urea, 0.2% Phenol Red, 0.02% NaN_3_, 0.01 mol/L pH 6.5 NaPO_4_ buffer). DNA was extracted from stomach homogenate using the QiaAMP DNeasy Blood and Tissue kit (Qiagen, Hilden, Germany) per the manufacturer's instructions. Extracted DNA was tested for the presence of the *H. pylori* VacA gene by PCR using the Takara PCR kit (Fisher, TAK RR001A) and primers VagA‐F (5‐CAATCTGTCCAATCAAGCGAG) and VagA‐R (5‐GCGTCAAAATAATTCCAAGG).^31^ PCR was run with an initial denaturation at 95°C for 3 minutes, followed by 35 cycles of 95°C for 1 minute, 52°C for 1 minute, 72°C for 1 minute, and a final extension at 72°C for 5 minutes. PCR products were visualized on a 1.5% agarose gel.

## 3 Results

### 3.1 Morphology, culturability, and metabolic activity of *H. pylori* in water

**Figure 1:**
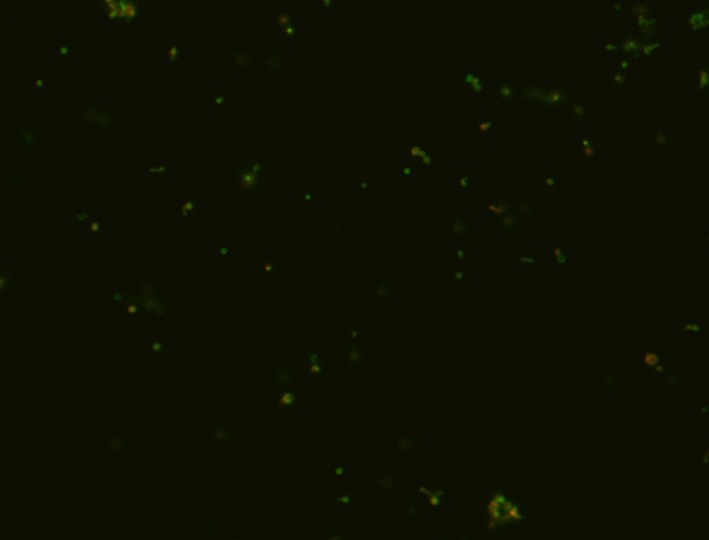
60× magnification of *Helicobacter pylori* suspension in water after 8 d of incubation at room temperature. Green cells are membrane intact, and red cells have membrane damage. The predominant form was coccoid

In all experiments, there was a complete loss of culturability of *H. pylori* 2‐3 days after initial inoculation into water. Despite being nonculturable, cells were still found to be membrane intact via Live/Dead staining 8 days after inoculation into water (Figure 1).

**Figure 2:**
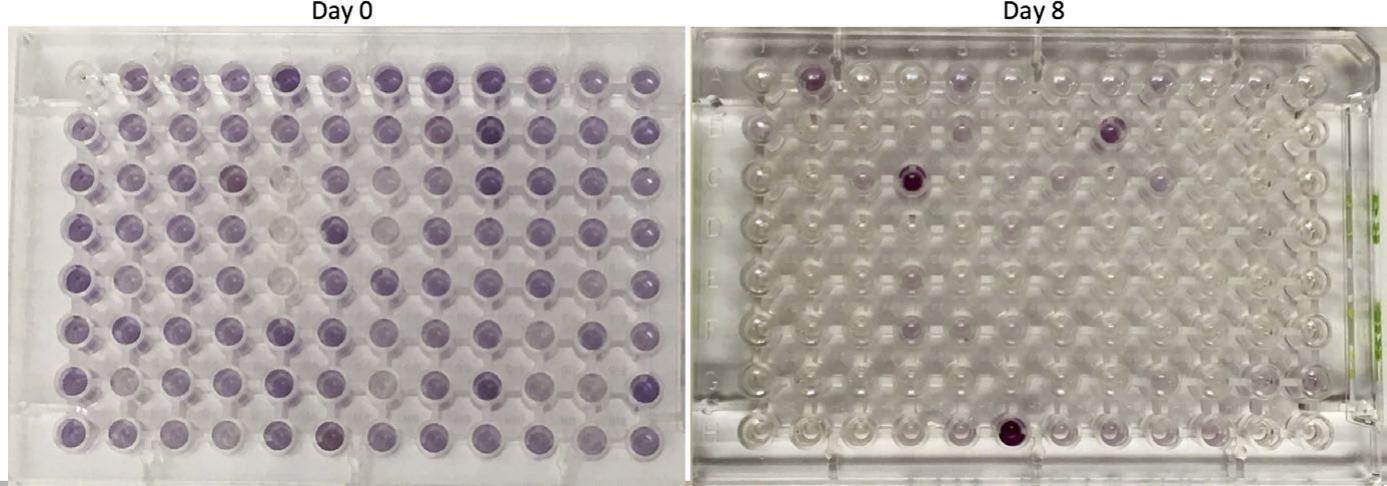
PM1 plates of Day 0 (viable and culturable) and Day 8 *Helicobacter pylori* cells (VBNC). Each well contains a different carbon source, and wells with a purple color change indicate that the carbon source was being used. Viable culturable *H. pylori* utilized a much wider variety of carbon sources than the VBNC *H. pylori*

The VBNC *H. pylori* cells also induced color changes in the Biolog PM1 panels at each time point, respectively (Figure 2). This suggests that the cells were metabolically active, as they were metabolizing the carbon sources in each well. The cells in the viable, culturable state utilized many more carbon sources than any of the cells in the VBNC state. No differences in metabolic activity were seen between VBNC cells on days 3, 4, 7, and 8 (data not shown).

### 3.2 Exposure to waterborne *H. pylori*

A cage was counted as infected if the following conditions were met: the quantitative culture plates had colonies with correct *H. pylori* morphology (small, round, and translucent), were positive for the rapid urease test, and were positive for PCR targeting the VacA gene. Cages were counted as positive if one or both mice in a cage were infected. If no mice were infected, then that cage was counted as negative. The results of the five exposure scenarios and the positive and negative controls are summarized in Table 2. Further, the mice dosed with SS1 via gavage were also not infected.

**Table 2:** Overview of experimental results

Experiment number	Average number of VBNC *Helicobacter pylori* cells per liter drinking water (range)	Average cumulative ingested dose per mouse (range)	Number of infected cages n/N (%)	Total number of infected mice n/N (%)	VacA PCR‐positive results n/N (%)
Experiment 1	10^9^	10^6^	0/20 (0%)	0/40 (0%)	0/40
Experiment 2	2.14E9 (1.15E9‐3.42E9)	5.33E7 (4.09E7‐6.91E7)	0/20 (0%)	0/40 (0%)	0/40
Experiment 3	2.22E9	5.44E6 (4.20E6‐6.19E6)	0/5 (0%)	0/10 (0%)	0/10
Experiment 4	7.49E9 (9.30E8‐2.04E10)	2.30E9 (1.75E9‐3.83E9)	0/50 (0%)	0/100 (0%)	0/100
Negative control	0	0	0/5 (0%)	0/10 (0%)	0/10
Positive control	4.80E9 (2.42E8‐2.04E10)	1.07E9 (8.45E8‐1.64E9)	5/5 (100%)	8/10 (80%)	8/10 (80%)

Results from viable but nonculturable *H. pylori* dosing experiments. While the positive control showed consistent levels of infection with previous studies, mice exposed to VBNC *H. pylori* showed no signs of infection.

The negative controls showed no signs of infection, and confirmed *H. pylori* cultures were recovered from 8 of 10 positive controls. None of the mice exposed to VBNC *H. pylori* showed any sign of infection, either via culture or PCR.

## 4 Discussion

We were unable to cause infection in mice with the VBNC form of SS1, either in drinking water or via gavage. Our inability to cause infection was surprising, given the known capacity of this strain to successfully infect mice,^26^, ^32^ our wide range of exposure scenarios, and our previously published study that showed that SS1 in water could infect mice in a dose‐dependent manner.^20^ In our previous study, 4 weeks of exposure to water spiked with 10^9^ CFU/L, 10^8^ CFU/L 10^7^ CFU/L, and 10^6^ CFU/L of *H. pylori* caused infection in 39 of 40, 33 of 40, four of 38, and one of 40 mice, respectively. The ingested cumulative doses are two‐ to 2000‐fold lower than those used in this current experiment, showing that SS1 is less infectious (or completely noninfectious) in the VBNC state than when viable and culturable. This suggests that *H. pylori* strains may be less infectious than when viable and culturable.

However, there are few dosing experiments in the literature that examine this phenomenon. She et al.^19^ found that 11 of 16 mice gavaged with VBNC *H. pylori* were infected compared to 14 of 16 gavaged with the same dose of viable, culturable *H. pylori*. Also using gavage to administer doses, Cellini et al.^27^ showed that eight of 20 mice were infected from VBNC *H. pylori* compared to nine of 20 with viable, culturable *H. pylori*. Both studies used strains that were recently isolated from clinical biopsies of patients with ulcers. Combined with our results from drinking water and gavage exposure to SS1, this suggests that different strains may differ in their ability to infect mice when in the VBNC state.

Our inability to cause infection could be due in part to the drinking water exposure route, which may affect the dose that reaches the stomach compared to gavage methods. Gavage directly inoculates the stomach with a large bolus of bacteria, while drinking water contains comparatively lower doses and must go through the mouth and esophagus before reaching the stomach, which may result in bacterial losses along the way. While this may affect our results, our total cumulative doses—especially in experiment 5—were comparable to (or higher than) the doses reported in previous studies (10^8^‐4*10^8^ CFU/dose). Further, our gavage experiments showed no signs of infection either. Finally, our previous study in which we administered viable, culturable *H. pylori* to mice in drinking water found relatively similar dose/response rates as other studies that were carried out with gavage.^20^

### 4.1 Limitations and public health implications

As with any animal study, we cannot be certain that our results accurately reflect what would occur with human exposure. As *H. pylori* is a human pathogen, it is possible that the VBNC form is more infectious in humans than in mice. Further, we only exposed mice to one strain of *H. pylori*, and it is possible that other strains would be more infectious in the VBNC state than SS1, as has been seen in other published papers in the literature.^19^, ^27^ Despite our large sample sizes and high doses, our inability to infect mice with VBNC *H. pylori* via drinking water suggests that VBNC SS1 in water is not infectious in mice. This may reflect the strain that we used, the route of exposure, or may simply mean that we did not account for some crucial piece of the puzzle that is yet unknown about the transmission of *H. pylori* via water. The genetic variability of *H. pylori* strains is vast,^33^ so it may be possible that some strains lack the capability to persist in water, but instead are transmitted only via other exposures, such as person‐to‐person or fecal‐oral routes.^4^

### 4.2 Future directions

Examining different strains of VBNC *H. pylori* in these exposure scenarios would give insight into the trade‐offs of survival and infectivity associated with the VBNC state. Further, investigating the distributions of VBNC vs viable, culturable *H. pylori* populations in the natural environment would provide a better understanding of the infectivity of the various forms of *H. pylori*. Such experiments would allow for more accurate risk assessments of *H. pylori* in water, as it is very likely that multiple strains and forms of *H. pylori* are present in contaminated drinking or surface water sources.

## 5 Conclusions

We found that mice exposed to VBNC SS1 *H. pylori* via drinking water were not infected, despite the various exposure scenarios (immunocompromised, high doses) that might have promoted infection with VBNC bacteria. While other studies that have used viable, culturable SS1 to successfully infect mice via gavage and drinking water, our results suggest that VBNC SS1 is either not infectious (or potentially has greatly reduced infectivity). Future studies could examine different *H. pylori* strains in similar exposure scenarios to compare the relative infectivity of the VBNC vs the viable, culturable state, which would help inform future risk assessments of *H. pylori* in water.

## Disclosures of Interests

The authors declare no conflict of interests.
